# A Troubling Diagnosis of Verrucous Squamous Cell Carcinoma (“the Bad Kind” of Keratosis) and the Need of Clinical and Pathological Correlations: A Review of the Literature with a Case Report

**DOI:** 10.1155/2011/370605

**Published:** 2010-10-25

**Authors:** A. Santoro, G. Pannone, M. Contaldo, F. Sanguedolce, V. Esposito, R. Serpico, L. Lo Muzio, S. Papagerakis, P. Bufo

**Affiliations:** ^1^Section of Anatomic Pathology and Cytopathology, Department of Surgical Sciences, University of Foggia, Viale L Pinto 1, 71100 Foggia, Italy; ^2^Department of Odontostomatological, Orthodontical and Surgical Sciences, Second University of Naples, Via De Crecchio 1, 80138 Naples, Italy; ^3^Section of Oral Pathology, University of Foggia, Viale L Pinto 1, 71100 Foggia, Italy; ^4^Division of Head and Neck Surgery and Oncology, Department of Otolaryngology, University of Michigan Medical School, Ann Arbor, MI 48109, USA

## Abstract

Verrucous carcinoma (also known as Ackerman tumor) is an uncommon exophytic low-grade well-differentiated variant of squamous cell carcinoma. This neoplasm typically involves the oral cavity, larynx, genitalia, skin, and esophagus. It is well known for its locally aggressiveness and for its clinically slow-growing behaviour with minimal metastatic potential. Verrucous carcinoma of oral cavity is so closely aligned with the use of snuff and chewing tobacco that it has been called the “snuff dipper's cancer”. Recent studies have proved the role of HPV. The typical clinical presentation of oral verrucous carcinoma has long been known, as its remarkably innocuous appearance and biological behaviour. In this work, we report a review of the scientific literature and describe a troublesome case of oral verrucous cancer.

## 1. Introduction

Verrucous carcinoma (VC) was defined by Ackerman in 1948 [1] as a diagnostically problematic squamous cell neoplasia involving lip, oropharyngeal, and laryngeal mucosa. As a result, this neoplasm was named as “Ackerman's tumor.” VC is strongly associated with the chronic use of tobacco or with the practice of chewing betel nuts [2]. According to recent studies, human papillomavirus could have a potential role in the tumoral development and progression, although this topic is still under discussion [3].

This uncommon lesion, in its pure form, can be considered a disease of later life, typically occurring in the seventh-eighth decades, with a strong male predominance. In the south-eastern USA women were particularly affected because of their historically common practice of snuff dipping. In the head and neck district, VC most frequently involves the oral cavity, where it commonly arises from buccal mucosa and lip. It is considered a slowly growing neoplasm that can reach considerable size before being brought to medical attention [4]. It appears as a papillary nonulcerated gray-white or red mass with a very broad base of attachment. This heavily keratinized, well differentiated variant of squamous cell carcinoma shows warty-like aspects and lacks conventional cytologic findings of atypia, exhibiting only locally invasiveness and no metastatic potential [5]. 

We report a case of oral VC, referring all the diagnostic difficulties occurring in the clinicopathological examination.

## 2. Case Report

**Figure 1:**
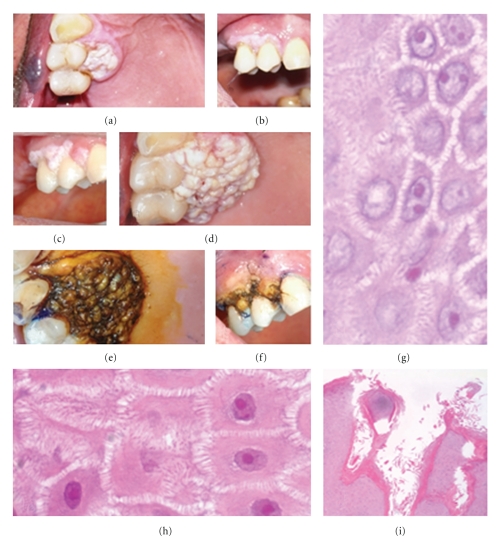
Clinical findings and histopathological details. Clinical aspects at the first observation: a verrucous, exophytic mass, about 1 cm in diameter, localized in the hard palate (a) at the upper right premolars and extending to the vestibular side of the gingival (b). Clinical findings 15 days later: in two weeks the lesion grew larger and triplicated its size (c, d). Staining by toluidine blue and Lugol was performed to guide incisional biopsies (e, f). Histological details of an area with mild basal cytological atypia: vesicular nuclei with prominent eosinophilic nucleoli ((g, h); Haematoxylin and Eosin, ×40). Superficial “church pinnacles” dyskeratosis ((i); Haematoxylin and Eosin, ×10).

During an oral examination a 77-year-man showed a whitish verrucous, exophytic mass, about 1 centimetre in diameter, localized in the hard palate at the upper right premolars and extending also to the vestibular side of the gingiva. He referred to have noticed the lesion two weeks before. It was painless and not bleeding. He quit smoking since 50 years, and he is currently affected by arterial hypertension in pharmacological treatment. The lesion was first stained by toluidine blue and Lugol stainings, and then incisional biopsies were performed at the observation time and 15 days later. In two weeks the lesion grew larger and triplicated its size (Figure 1). The histopathological result was of “oral verrucous hyperplasia.” Nevertheless, the lesion has continued to grow, and a total excision with an osseous box and “en bloc” avulsion of involved teeth (1.3-1.4-1.5) were done. According to the clinical behaviour of the lesion, the correct diagnosis was of VC, as confirmed by some histopathological features showed by the second biopsy. Actually, 2 weeks after the surgical treatment, the patient showed a similar lesion close to the distal excision margin, on the palatal side.

## 3. Gross and Microscopic Findings

The first incisional biopsy was histopathologically assessed as verrucous hyperplasia, showing (1) proliferation of cutaneous epithelium, resulting in variable epithelial thickness, (2) verrucous exophytic component, (3) smoothly contoured, though irregular/asymmetrical and not pushing, (4) no evidence of frank invasion, and (5) mild dysplasia of deep keratinocytic layers (Figure 1).

**Figure 2:**
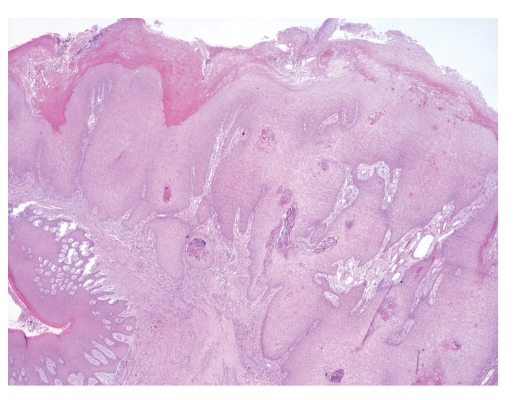
Microscopic aspects supporting diagnosis of verrucous carcinoma. Note the broad pushing blunt squamous epithelial downgrowths that are diagnostic of verrucous carcinoma (Haematoxylin and Eosin, ×2).

The definitive surgical specimen shows a tan mass, with a papillary wart-like surface and sharp borders. Histologically, pathologists confirmed the clinic diagnosis of VC, on the base of these features: dense superficial keratinisation, dyskeratosis, minimal cytological atypia, pushing margins without a frank infiltration (Figure 2), nor vascular or perineural invasion, and prominent lymphoplasmacytic infiltrate at the base of the lesion.

## 4. Review of the Literature and Conclusion

The clinico-histo-pathological diagnosis of VC is often exclusionary and extremely difficult [6]. The precise histological categorisation of mucosal lesions that include an exophytic growth component is a difficult and often encountered experience. It has been reported that multiple biopsies over several years are often required before diagnostic histological features supporting an appropriate interpretation are identified [7]. 

The term “verrucous” was applied for lesions showing a keratotic exophytic surface composed of sharp or blunt epithelial projections with keratin-filled invaginations (plugging), but without obvious fibrovascular cores. The histological features of VC, for example, verrucous surface and “elephant feet-” like downgrowth seeming to compress the underlying connective tissue and typically showing minimal or absent cytological atypia, are widely known [8]. The diagnostic difficulties fall into different categories. Lesions with a “verrucous” surface may be VC or show the conventional invasive pattern. The latter represents an “SCC with an exo-endophytic growth pattern.” Often the invasion can be lacking in incisional biopsies, and it is not possible to exclude an underlying conventional carcinoma. Distinction from classical squamous cell carcinoma is a frequent problem also for clinicians because of the extensive nature of the lesion mimicking an invasive cancer [8]. An important help could be offered by molecular approaches. VC shows the characteristics of cell kinetics that are similar to those of normal epithelium and not to conventional squamous carcinoma. S-phase is confined to basal layer, unlike the invasive cancer. By flow cytometry, VC is a diploid lesion; on the contrary, the conventional squamous cancer often shows aneuploidy and genomic instability [9]. 

In superficial biopsies without an obvious invasive growth, the bland benign appearing cytology may, also, induce to an erroneous diagnosis of benign squamous proliferation [1]. This problem was the same troubling question that affected Dr. Ackerman in the original study of this type of lesion. An anecdote that he related during his extensive travels dealt with his early experience at the Ellis Fischel Cancer Center and a patient who had multiple biopsies of an oral cavity lesion which Dr. Ackerman had repeatedly labelled as “keratosis.” He personally examined the patient and confronted him with an obviously invasive lesion extending through the oral cavity and involving the skin of the cheek. The pathologist, when a surgeon asked him the specific “nature” of a problematic lesion arisen on the oral cavity, replied as “The bad kind!” to confirm the difficulty in the diagnosis of a VC on incisional biopsies [1]. Because it is cytologically benign, besides the focal basal cell nuclear hyperchromatism, distinction from VC and verrucous hyperplasia (VH) cannot be based only on cytologic features [8, 10]. The final diagnosis of VC can eventually depend on identifying, as in our case, the characteristic peripheral buttressing and shouldering. Consideration of the clinical history is important in preventing misdiagnoses. It should be also noted that some authors considered VH as a continuum of lesions simultaneously occurring with or metachronously evolving into VC and/or conventional SCC [8, 10]. Anyway, it is essential that the pathologist alerts the clinician to the progressive nature of the lesion and recommends complete excision or close followup and rebiopsy [8, 10]. In sum differential diagnosis, VC should be analysed regarding (a) conventional SCC, especially with those SCC showing “verrucoid” features, (b) proliferative verrucous leukoplakia (PVL), (c) reactive keratosis and epithelial hyperplasia, (d) pseudoepitheliomatous hyperplasia, (e) verruca vulgaris, and (f) keratoacanthoma when verrucous carcinoma affects cutaneous sites. In particular it is mandatory to rule out hybrid carcinoma including VC and conventional SCC. Hybrid carcinomas should be staged and managed as conventional SCC because their metastatic potential, contrary to classical VC, shows excellent prognosis following complete surgical removal in the early stages. 

Finally we report the controversy regarding the role and the effect of radiation therapy on VC [11]. Surgical resection remains the choice treatment for this neoplasm. Neck dissection is not indicated for any pure VC, given the absence of nodal metastases; cervical adenopathy may be associated with VC, representing reactive changes and not metastatic disease. The literature supports the concept that radiotherapy is contraindicated in the treatment of VC for the occurrence of Radiation-induced anaplastic transformation, manifesting 2 to 8 months following the therapeutic cycle [11]. As the literature is confusing, we retain that radiotherapy could be used only in selected clinical settings, when surgery is not possible.

This paper has identified the common pitfalls during routine assessment of surgical or bioptical material: the pathologically underdiagnosed cases (diagnosis of keratosis or VH, with synchronous or metachronous aggressive pattern of growth), the clinically undertreated (PVL and VC, without a histological diagnosis of dysplasia), and the surgically overtreated cases (VC treated as a conventional invasive SCC, with demolitive surgical resection and useless lymphadenectomy). The final pathological diagnosis should involve staff from multiple disciplines. We realize that a correct diagnosis is founded on the precise comparison and integration of all the results and not on the isolated valuation of the different findings. In this context the importance of close cooperation with clinicians is essential.
